# Bio‐absorbable mesh and early positron emission tomography avidity: implications in oncological surveillance

**DOI:** 10.1111/ans.70043

**Published:** 2025-02-28

**Authors:** Ernest Cheng, Mina Sarofim, Amit Sarkar, Assad Zahid, Andrew Gilmore

**Affiliations:** ^1^ Department of Colorectal Surgery Liverpool Hospital Liverpool New South Wales Australia; ^2^ St George and Sutherland Clinical School University of New South Wales Kogarah New South Wales Australia; ^3^ School of Medicine Western Sydney University Liverpool New South Wales Australia; ^4^ Faculty of Medicine and Health Sciences Macquarie University Hospital Macquarie Park New South Wales Australia

A 28‐year‐old male with locally advanced rectal cancer involving bladder and right pelvic sidewall underwent total neoadjuvant therapy (TNT) followed by total pelvic exenteration. The procedure included abdominoperineal resection, cystoprostatectomy, urethrectomy and coccygectomy. Reconstruction of the pelvic floor was performed using a bio‐absorbable mesh (Gore® Bio‐A® Tissue Reinforcement), which was fashioned into a cup shape and reinforced with an overlying omental graft.

**Fig. 1:**
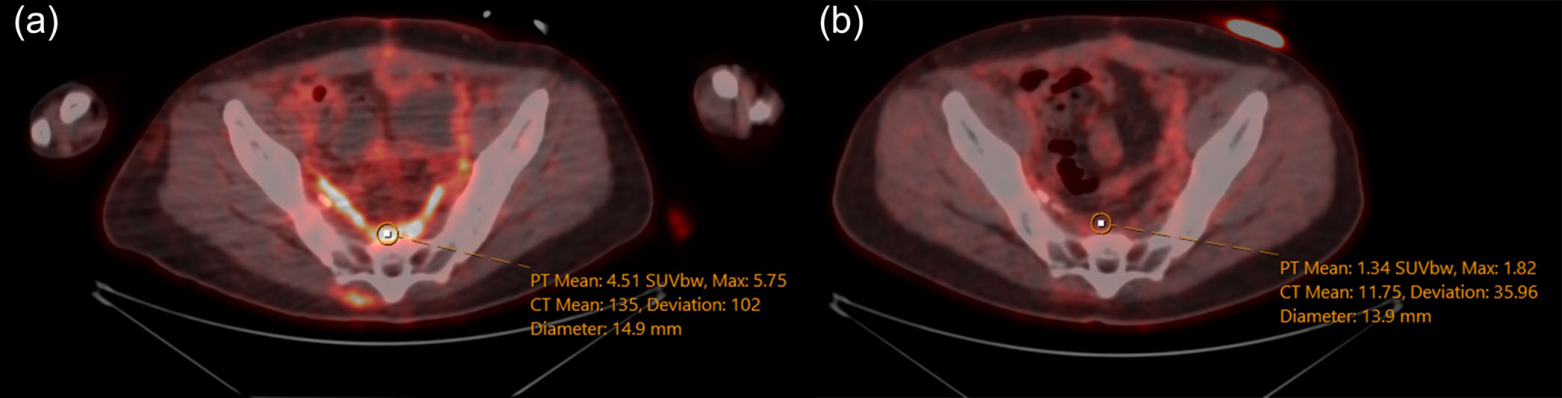
(a) PET/CT fusion axial view performed 1 month postoperatively demonstrated high FDG avidity in the shape of the placed bio‐absorbable mesh. (b) PET/CT fusion axial view performed 9 months postoperatively showing significantly decreased FDG avidity.

**Fig. 2:**
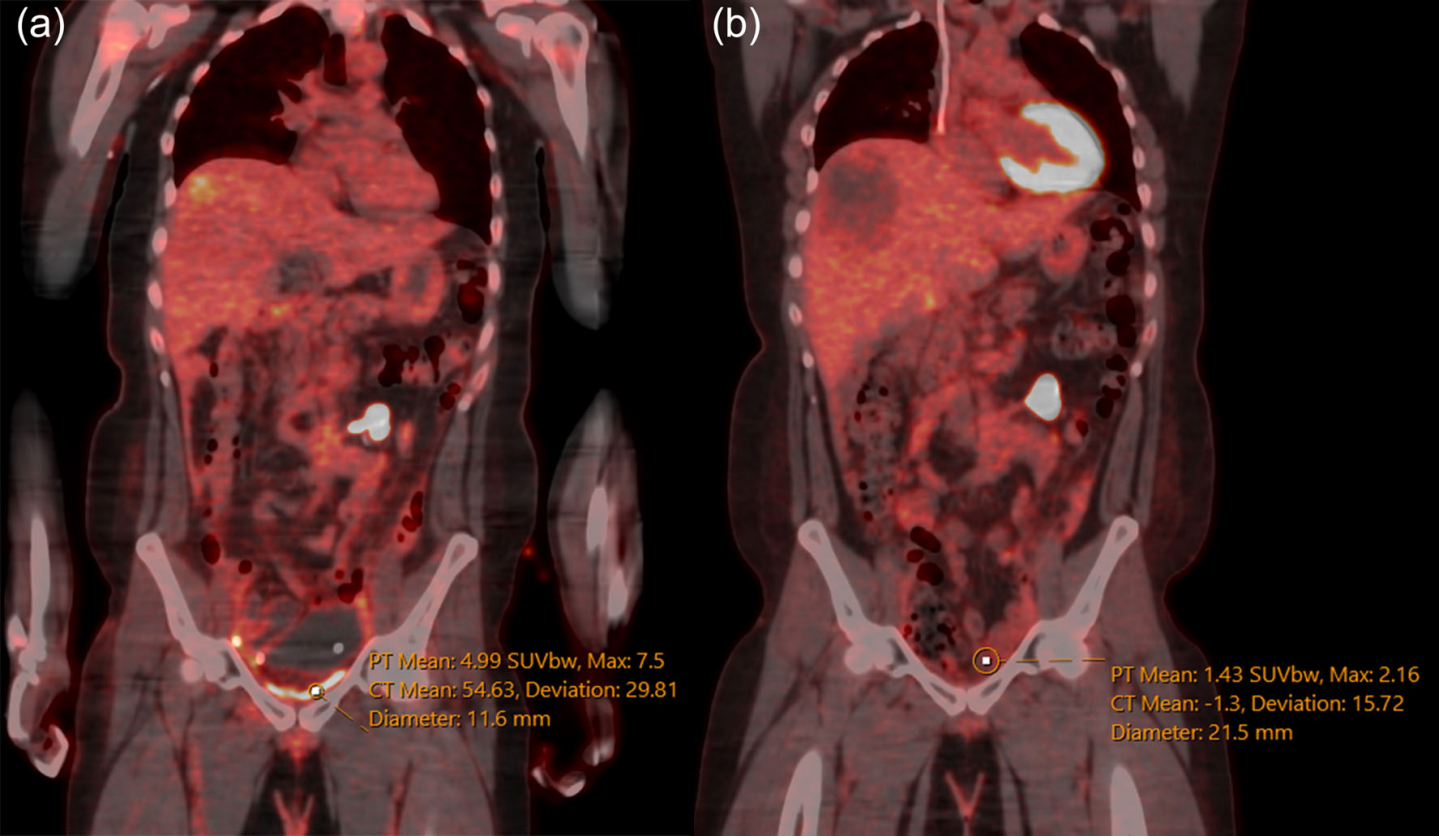
(a) PET/CT fusion coronal view at 1 month postoperatively. (b) PET/CT fusion coronal view at 9 months postoperatively.

One month postoperatively, computerized tomography (CT) revealed a new liver lesion prompting early a positron emission tomography (PET) scan. The PET scan demonstrated diffuse fluorodeoxyglucose (FDG) uptake in the pelvis up to a standardized uptake value (SUV) of 7.5. This was presumed to be related to the bio‐absorbable mesh rather than disease recurrence. A subsequent PET scan 9 months later demonstrated minimal FDG uptake in the same region, with a SUV of 2.16 (Figs 1 and 2).

This case demonstrates the post‐operative FDG avidity associated with bio‐absorbable mesh. It highlights potential diagnostic challenges associated with the use of bio‐absorbable mesh in patients that may undergo early PET for cancer surveillance. Placement of mesh to reinforce the pelvic repair is a common practice in pelvic exenterations to minimize complications of the empty pelvis, such as perineal hernia, pelvic sepsis, entero‐perineal fistulas and bowel obstructions.^1^ Gore® Bio‐A® is a biosynthetic web scaffold consisting of synthetic polymers designed to gradually absorb over a 6‐month period and preferred in contaminated environments.^2^ Bio‐A was designed to recruit an inflammatory response during mesh absorption there by allowing it to be replaced by the patient's own connective tissue.^3^

PET scans are widely used for oncological follow‐up because of their high sensitive in detecting malignancy, but they have low specificity, particularly in the setting of concurrent inflammation. FDG avidity reflects metabolic activity in tissue, and both tumours and inflammation can present similarly.^4^ The duration of FDG avidity following surgical mesh placement is not well‐characterized. Davidson *et al*. observed variability in the persistence and intensity of FDG uptake for synthetic mesh used in hernia repairs, with some cases exhibiting persistent uptake for as long as 17 years.^5^

Our case uniquely illustrates the timeline for the resolution of FDG avidity in bio‐absorbable mesh. Bio‐A® mesh, which has a targeted absorption period of 6 months, was consistent with the absence of FDG avidity observed on PET scan 9 months postoperatively.^2^ This also raises a cautionary note for those interpreting PET scans during the first initial months following the placement of biosynthetic absorbable mesh. Currently, no other cases have demonstrated relationship between Bio‐A® and PET avidity in this context. False positive FDG avidity in the postoperative period is well recognized in the literature.^6^, ^7^ The inflammatory response to surgery, including formation of granulation tissue and interactions between key inflammatory cascades transiently increases FDG update.^7^ The optimal timing of performing a PET scan after surgery relies on clinical indication, type of cancer, and treatment received. Recommendations typically range between 1 and 3 months postoperatively.^6^, ^7^

We emphases the importance of multidisciplinary input among surgeons, radiologists, and oncologists in the interpretation of postoperative PET scans to ensure optimal evaluations. Surgeons should clearly document the operative mesh size, shape and placement, as this information is crucial for image interpretation and guide subsequent oncological investigations and management. Comparing serial imaging and combining PET scans with CT and magnetic resonance imaging studies can further aid in distinguishing true malignant recurrence from bio‐absorbable mesh related PET avidity.
